# Complete genome sequence of the Radiation-Resistant bacterium *Rubrobacter radiotolerans* RSPS-4

**DOI:** 10.4056/sigs.5661021

**Published:** 2014-03-29

**Authors:** C. Egas, C. Barroso, H.J.C. Froufe, J. Pacheco, L. Albuquerque, M.S. da Costa

**Affiliations:** 1 Next Generation Sequencing Unit, Biocant, Biotechnology Innovation Center, Cantanhede, Portugal; 2 Center for Neuroscience and Cell Biology, University of Coimbra, 3004-517 Coimbra, Portugal; 3 Department of Life Sciences, University of Coimbra, Coimbra, Portugal

**Keywords:** *Rubrobacter radiotolerans*, radiation-resistance, gram positive, genome sequence, 454 sequencing

## Abstract

*Rubrobacter radiotolerans* strain RSPS-4 is a slightly thermophilic member of the phylum “*Actinobacteria*” isolated from a hot spring in São Pedro do Sul, Portugal. This aerobic and halotolerant bacterium is also extremely resistant to gamma and UV radiation, which are the main reasons for the interest in sequencing its genome. Here, we present the complete genome sequence of strain RSPS-4 as well as its assembly and annotation. We also compare the gene sequence of this organism with that of the type strain of the species *R. radiotolerans* isolated from a hot spring in Japan. The genome of strain RSPS-4 comprises one circular chromosome of 2,875,491 bp with a G+C content of 66.91%, and 3 circular plasmids of 190,889 bp, 149,806 bp and 51,047 bp, harboring 3,214 predicted protein coding genes, 46 tRNA genes and a single rRNA operon.

## Introduction

*Rubrobacter radiotolerans* strain RSPS-4 is a slightly thermophilic actinobaterium isolated from a hot spring in central Portugal [1]. Species of the genus *Rubrobacter* are extremely resistant to ionizing radiation [1-4]. The type strain of *Rubrobacter radiotolerans* P-1^T^ (DSM 5868^T^, JCM 2153^T^) from Japan and strain RSPS-4 are two of the most resistant organisms to ionizing radiation, more so than the archetypal *Deinococcus rediodurans* strain R1 (DSM 20539^T^, JCM 16871^T^) [5]. Both *R. radiotolerans* strains have a sigmoid shaped survival curve on a dose-response irradiation curve up to 25 kGy and a shoulder dose of 5.7 kGy, with a 37% survival of 7.6 and 9.0 for the type strain of *R. radiotolerans* and strain RSPS-4 [1].

Microbial resistance to ionizing radiation is rather intriguing since gamma radiation is restricted to locations contaminated with nuclear waste, while natural environments with high doses of gamma radiation are not known in the biosphere. One hypothesis is that radiation resistance is related to desiccation resistance in some organisms that do not produce spores [6]. While the species of *Deinococcus* are the preeminent extremely radiation-resistant organisms [7], other radiation-resistant microbes are isolated from very diverse environments and are classified in taxa that belong to different phyla and domains, suggesting that this characteristic evolved independently and in response to other environmental challenges, possibly desiccation or reactive oxidative stress (ROS). Radiation-resistant organisms have been described from archaea such as *Thermococcus gammatolerans* [8] to bacteria, such as *Deinococcus* [9] and *Truepera* [10], both of which belong to the phylum *Deinococcus-Thermus*, *Actinobacteria* of the genera *Rubrobacter* [1] and *Kineococcus* [11], *Proteobacteria* of the genera *Methylobacterium* [12] and *Acinetobacter*, *Sphingobacteria* of the genus *Hymenobacter* [9] and cyanobacteria of the genus *Chroococcidiopsis* [13].

*Deinococcus radiodurans* has been the most studied bacterium for radiation resistance mechanisms [14-20], however, no single key factor has been identified to explain its resistance and it is now hypothesized that the ability to recover from high doses of irradiation results from a combination of several mechanisms. DNA repair systems were the first studied resistance mechanisms, but *D. radiodurans* contains a set of enzymatic repair systems similar to radiation-sensitive bacteria such as *Shewanella oneidensis* and *Pseudomonas putida* [15,21] and such systems are thus insufficient *per se* to justify the almost error-free reassembly of the irradiated *D. radiodurans* genome [22]. Recent findings shifted the focus from DNA repair to antioxidant protein protection as a main resistance mechanism [15,23]. Several studies showed that irradiated cells maintained enzymes protected from oxidative stress and thus available for an efficient repair of DNA lesions [24-27].

Here, we describe the complete sequencing and annotation of the genome of *R. radiotolerans* RSPS-4, identify genes involved in the main repair pathways for DNA, the synthesis of compatible solutes that could be involved in the protection of enzymes and in the response to oxidative stress, and analyze the differences between strain RSPS-4 and the type strain P-1^T^ (DSM 5868^T^, JCM 2153^T^). This genome will help understand the genetic basis for radiation resistance mechanisms and provide data for broader comparative studies with other radiation resistance bacteria.

## Classification and features

*Rubrobacter* belongs to the phylum “*Actinobacteria*” formerly known as the high G+C Gram-positive bacteria. The genus comprises four species, three of which, were isolated from thermal environments, namely *Rubrobacter xylanophilus* recovered from the thermally polluted runoff from a carpet factory in the United Kingdom [2]; *Rubrobacter radiotolerans* [3] isolated from a hot spring in Japan after gamma-irradiation of the water sample [28] and *Rubrobacter taiwanensis* isolated from non-irradiated samples from Lu-Shan hot spring in the central region of Taiwan [4]. The mesophilic species *Rubrobacter bracarensis*, was isolated from a green biofilm covering the biodeteriorated interior walls of a Church at Vilar de Frades, in Portugal [29], but is not known to be gamma-, UV- or desiccation-resistant.

**Figure 1:**
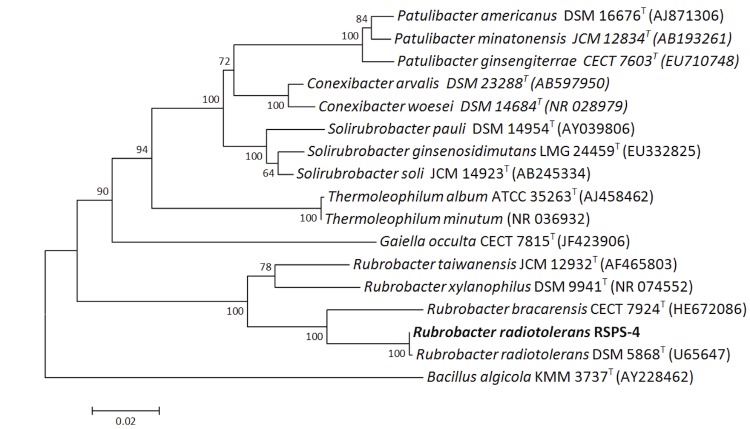
Phylogenetic tree showing the position of *Rubrobacter radiotolerans* strain RSPS-4 with other organisms within the subclass *Rubrobacteridae*. The tree was inferred from 1,301 aligned characters of the 16S rRNA sequences of different species using the Neighbor-Joining method [30]; bootstrap values are based on 1,000 replicates [31]. The evolutionary distances were calculated using the Jukes-Cantor method [32]. Analysis was carried out with MEGA6 [33]. *Bacillus algicola* KMM 3737^T^ (AY228462) was used as an outgroup.

Phylogenetic analysis of 16S rRNA gene sequences indicates that the species of the genus *Rubrobacter* belong to the monogeneric family *Rubrobacteraceae* (Figure 1), which along with the genera of the families *Solirubrobacteriaceae*, *Thermoleophilaceae, Conexibacteraceae, Patulibacteraceae* and *Gaiellaceae* form deep-branching lineages of the subclass *Rubrobacteridae* of the phylum “*Actinobacteria*” [34]. The species *R. radiotolerans* is most closely related to *R. bracarensis* [29].

The *Rubrobacter radiotolerans* strain RSPS-4 was isolated from a hot spring runoff at São Pedro do Sul, in central Portugal, after irradiation of the water sample with a cobalt-60 source at a dose of 8.06 kGy and a rate of 1.5kGyh^-1^ [1]. The hot spring runoff had a temperature of 50ºC and a pH of 8.9. The strain grew optimally at 45ºC in medium containing no added NaCl but was able to grow in medium containing 6% NaCl (w/v), degraded hide powder azure, gelatine, hippurate, arbutin and esculin, was cytochrome oxidase, catalase and β-galactosidase positive and produced nitrite from nitrate. The fatty acid composition of RSPS-4 was dominated by the unique 12-methyl-16:0 (70% of the total) with lower amounts of 4-methyl-18:0 (15.7%-17.8% of the total fatty acids). The 16S rRNA gene sequence of strain RSPS-4 is similar to the sequence of the type strain of *R. radiotolerans* DSM 5868^T^ [1], only differing in 2 positions. Classification and general features of *Rubrobacter radiotolerans* RSPS-4 are shown in Table 1. The strain was deposited at the Spanish Type Culture Collection (CETC) with the code CECT 8386.

**Table 1:** Classification and general features of *Rubrobacter radiotolerans* RSPS-4 according to the MIGS recommendations [35].

**MIGS ID**	**Property**	**Term**	**Evidence code**
		Domain *Bacteria*	TAS [36]
		Phylum *Actinobacteria*	TAS [37]
		Class *Actinobacteria*	TAS [38]
		Subclass *Rubrobacteridae*	TAS [36-40]
	Current classification	Order *Rubrobacterales*	TAS [38,40,41]
		Family *Rubrobacteraceae*	TAS [38-40]
		Genus *Rubrobacter*	TAS [42,43]
		Species *Rubrobacter radiotolerans*	TAS [42,43]
		Strain RSPS-4	
	Gram stain	Positive	TAS [1,3]
	Cell shape and pigmentation	Pleomorphic rod shaped; red pigmented, Pink colonies	TAS [1,3]
	Motility	Non-motile	
	Sporulation	Does not produce spores	TAS [3]
	Temperature range	30-55ºC	TAS [1]
	Optimum temperature	45ºC	TAS [1]
	Carbon source	Organic carbon compounds	
	Energy source	Organic carbon compounds	
	Terminal electron receptor	O_2_, nitrate	
MIGS-6	Habitat	Hot springs	TAS [1,3]
MIGS-6.3	Salinity	<Than 0.2% NaCl	TAS [1]
MIGS-22	Oxygen	Aerobic	TAS [1,3]
MIGS-15	Biotic relationship	Free living	TAS [1,3]
MIGS-14	Pathogenicity	None	
MIGS-4	Geographic location	São Pedro do Sul	TAS [1]
MIGS-5	Sample collection time	1999	TAS [1]
MIGS-4.1	Latitude	40° 44' 22.09'' N	
MIGS-4.2	Longitude	8° 5' 32.47'' W	TAS [1]
MIGS-4.3	Depth	Surface hot spring	

Evidence codes - TAS: Traceable Author Statement (i.e., a direct report exists in the literature); NAS: Non-traceable Author Statement (i.e., not directly observed for the living, isolated sample, but based on a generally accepted property for the species, or anecdotal evidence). These evidence codes are from of the Gene Ontology project [44].

## Genome sequencing information

### Genome Project History

*R. radiotolerans* strain RSPS-4 was selected for sequencing based on its extremely high resistance to ionizing radiation. The complete genome sequence is available from GenBank. Sequencing was performed at Roche Diagnostics GmbH, Penzberg, finishing and annotation were performed by Biocant. A summary of the project is shown in Table 2.

**Table 2:** Genome sequence project information

**MIGS ID**	**Property**	**Term**
MIGS-31	Finishing Quality	Finished
MIGS-28	Libraries Used	1 GS DNA Standard Library for 454 Pyrosequencing
MIGS-29	Sequencing Platforms	454 GS20 and 3500/3500XL Genetic Analyzer
MIGS-31.2	Fold Coverage	23× Pyrosequence and Sanger
MIGS-30	Assemblers	454 Roche Newbler Assembler, Phrap and Consed.
MIGS-32	Gene Calling Method	Prodigal

### Growth conditions and DNA isolation

*Rubrobacter radiotolerans* strain RSPS-4 was grown on *Thermus* medium at 45ºC as described elsewhere [1]. DNA isolation was performed from a 1g pellet of a 24h, exponential growth phase culture using the Wizard Genomic DNA Purification Kit (Promega, Madison, USA) following the standard protocol, without modifications, for genomic DNA isolation of Gram positive bacteria, as recommended by the manufacturer.

### Genome sequencing and assembly

The genome of strain RSPS-4 was sequenced in the GS20 sequencing platform (Roche -454 Life Sciences) at Roche Diagnostics GmbH, Penzberg, Germany. A total of 3 PicoTiterPlates generated 889,098 pyrosequencing reads with an average length of 106 bp. These were assembled into 70 contigs, with an N50 of 120,613, using the Newbler assembler (Roche). The Phred/Phrap/Consed software package [45,46] was used for sequence assembly and quality assessment in the subsequent finishing procedures. The gaps were closed by an Optimized Multiplex PCR approach [47], where 156 Sanger sequences were produced and added to the 454 reads to produce a hybrid assembly with Phrap. Together, 454 and Sanger sequences provided a 23× coverage of the genome.

### Genome annotation

Structural and functional annotation has been performed using PGP (Prokaryotic Genome Prediction) an in-house developed pipeline. PGP used tRNAscan-SE [48], RNAMMer [49] and PILERCR [50] to predict non-coding genes and miscellaneous features. These features were then masked and the CDS predicted with Prodigal [51]. Furthermore, PGP automatically corrected the start position of each CDS based on the Geneprimp [52] algorithm. Functional annotation was carried out under PGP in InterProScan [53] against PFAM [54], TIGRFAM [55], Hamap [56], PIRSF [57], PRINTS [58], SMART [59], SUPERFAMILY [60], ProSite [61] databases and RPS-BLAST against COG database [62] The product name of each CDS was assigned using TIGRFAM, COG and PFAM annotation [63]. Those CDS that were not annotated with these databases were assigned as hypothetical proteins. The automatic annotation was followed by a round of manual curation to eliminate obvious overlaps by visualization of the obtained functional annotation with Artemis [64]. Possible mis-assemblies and mis-annotations were corrected by comparison to the genome of *Rubrobacter radiotolerans* DSM 5868^T^, assembly of 2011, produced by the US Department of Energy Joint Genome Institute. The genome sequence was completed in 2013 and presented for public access in 2014.

### Nucleotide sequence accession numbers

The genome sequence for *Rubrobacter radiotolerans* strain RSPS-4 was deposited at GenBank under the accession number XXXXXXX (Chromosome), XXXXXXX (Plasmid 1), XXXXXX (Plasmid 2) and XXXXXX (Plasmid 3).

## Genome properties

**Figure 2:**
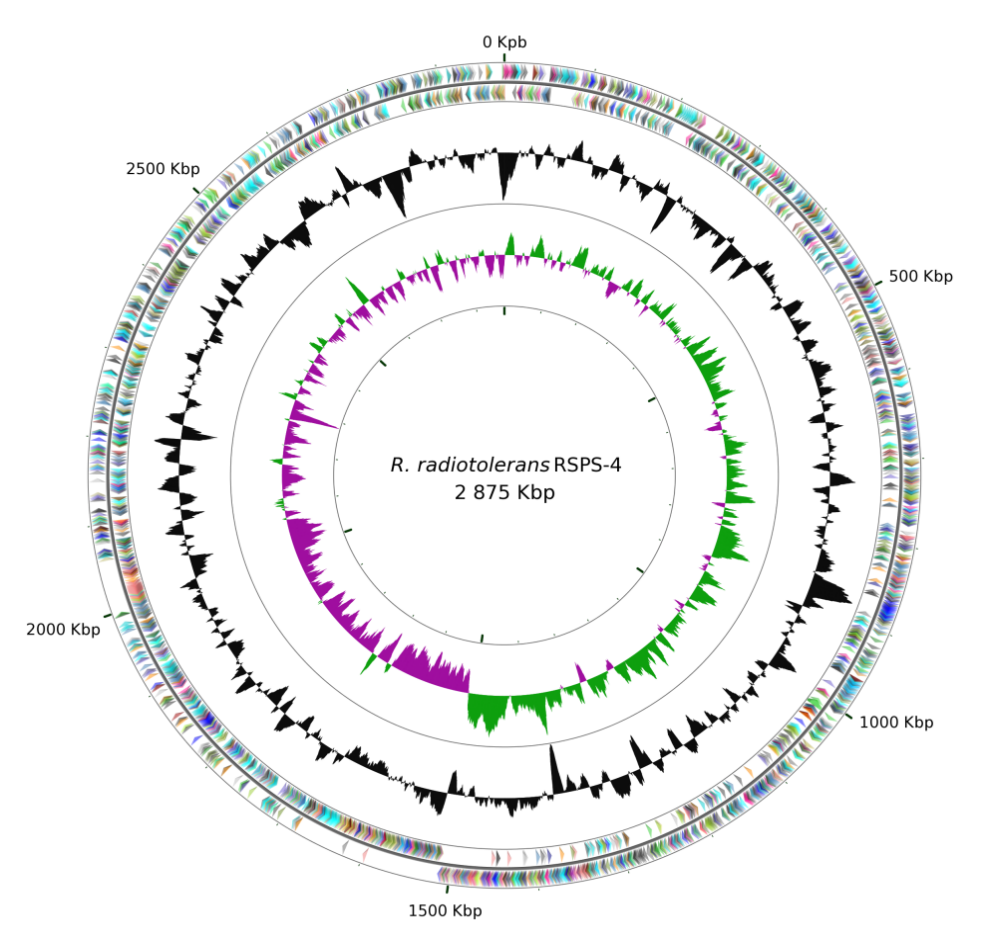
Circular representation of the chromosome of *R. radiotolerans* RSPS-4. From outside in, the outer two circles show genes on forward strand and reverse strand, colored by COG categories, the third circle shows the G+C% content plot (colored in black), and the inner circle the GC skew (green and purple). Graphics were created on the CGViewer Server [65].

**Figure 3:**
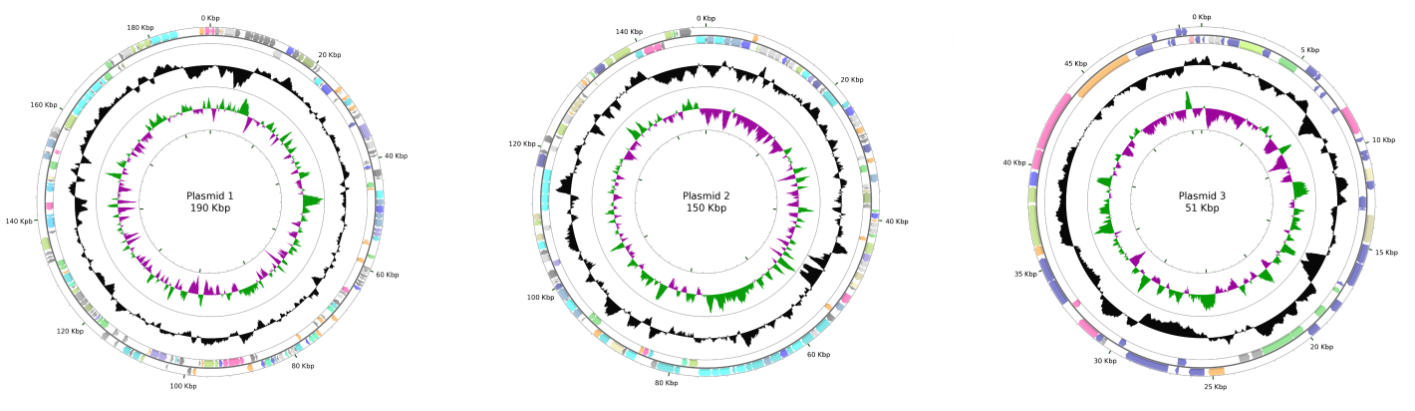
Circular representation of the plasmids of *R. radiotolerans* RSPS-4. From outside in, the outer two circles show genes on forward strand and reverse strand, colored by COG categories, the third circle shows the G+C% content plot (colored in black), and the inner circle the GC skew (green and purple). Graphics were created on the CGViewer Server [65].

The *Rubrobacter radiotolerans* strain RSPS-4 genome consists of one circular chromosome of 2,875,491 bp (G+C content 66.9%) (Figure 2) and three plasmids, with 190,889 bp (G+C content 65.66%), 149,806 bp (G+C content 66.48%) and 51,047 bp (G+C content 63.17%) (Figure 3). Of the 3,214 protein-coding genes predicted, 2,772 are located in the chromosome, 191 in plasmid 1, 137 in plasmid 2, and 59 in plasmid 3. The majority of the protein-coding genes, (2,646, 82%) were assigned a putative function while the remainder were annotated as hypothetical proteins. 46 tRNAs encoding all the 20 amino acids and 1 rRNA operon were annotated. The replication origin was set to the first nucleotide of the *dna*A gene. Genome properties are summarized in Table 3, Table S1 and Figure 2 and the distribution of genes into COGs functional categories is presented in Table 4.

**Table 3:** Genome Statistics

**Attribute**	**Value**	% of total
Genome Size (bp)	3,267,233	100
DNA Coding region (bp)	2,993,158	91.6
Chromosomal DNA G+C content (bp)	2,181,219	66.7
Extrachromossomal elements	3	
Total genes	3260	100
RNA genes	46	1.42
rRNA operons	1	
Protein-coding genes	3214	98.6
Genes with function prediction	2646	81.7
Genes assigned to COGs	2565	79.1
CRISPR repeats	2	

**Table 4:** Number of genes associated with the general COG functional categories.

**Code**	**Value**	**%age**	**Description**
J	139	4.46	Translation, ribosomal structure and biogenesis
A	-	-	RNA processing and modification
K	143	4.59	Transcription
L	118	3.79	Replication, recombination and repair
B	3	0.10	Chromatin structure and dynamics
D	30	0.96	Cell cycle control, mitosis and meiosis
Y	-	-	Nuclear structure
V	46	1.48	Defense mechanisms
T	121	3.89	Signal transduction mechanisms
M	143	4.59	Cell wall/membrane biogenesis
N	19	0.61	Cell motility
Z	-	-	Cytoskeleton
W	-	-	Extracellular structures
U	26	0.83	Intracellular trafficking and secretion
O	88	2.83	Posttranslational modification, protein turnover, chaperones
C	225	7.23	Energy production and conversion
G	162	5.20	Carbohydrate transport and metabolism
E	224	7.19	Amino acid transport and metabolism
F	75	2.41	Nucleotide transport and metabolism
H	131	4.21	Coenzyme transport and metabolism
I	128	4.11	Lipid transport and metabolism
P	136	4.37	Inorganic ion transport and metabolism
Q	37	1.19	Secondary metabolites biosynthesis, transport and catabolism
R	326	10.47	General function prediction only
S	245	7.87	Function unknown
-	649	20.84	Not in COGs

## Insights from the genome sequence

The genome sequence of strain RSPS-4 was analyzed for genes involved in recovery from ionizing radiation: DNA repair, oxidative stress response and compatible solute production. The key enzymes for the main DNA repair mechanisms were present in the genome, except for the non-homologous end joining, suggesting a shared set of DNA repair enzymes with other radiation-resistant bacteria. The pathways for oxidative stress response were also investigated but not all key enzymes were identified, suggesting that further studies are required in this bacterium to fully understand oxidative stress response and its role in radiation resistance. RSPS-4 possessed the four described pathways for trehalose production. Finally, the RSPS-4 genome sequence was compared to that of *R. radiotolerans* DSM 5868^T^ type strain. The two strains practically shared the same nucleotide sequence, the same gene order, orientation and synteny, however strain DSM 5868^T^ had extra segments, probably corresponding to putative prophages.

### DNA repair and associated systems

Gamma radiation induces the production of highly reactive oxygen radicals (ROS) in cells by ionizing water from several macromolecules. In DNA, ROS induce base modifications, DNA single-strand and double-strand breaks [66]. The latter are considered the most dangerous for survival and most difficult to repair. The *R. radiotolerans* RSPS-4 genome was analyzed for DNA repair pathways by searching genes involved in homologous recombination, single strand annealing (SSA), extended synthesis-dependent strand annealing or non-homologous end joining [67,68].

RSPS-4 strain encodes a set of essential genes for homologous recombination. All genes for the RecFOR pathway were detected, *rec*F (RradSPS_0004), *rec*R (RradSPS_0466), *rec*O (RradSPS_1511), and *rec*J (RradSPS_0780). RecA, the protein responsible for strand invasion and exchange, was encoded as a single copy (RradSPS_1428). Genes encoding the branch migration and resolution of Holliday junction proteins RuvA (RradSPS_1317), RuvB (RradSPS_1318), RuvC (RradSPS_1316), and RecG (RradSPS_1377) were detected, as were the homologs of the genes encoding the SbcD (mre11) (RradSPS_2355), and SbcC (Rad50) (RradSPS_2356) proteins. The gene coding for the RecX (RradSPS_1429) protein, which acts as a negative regulator of RecA, was also present.

The genes encoding proteins involved in extended synthesis dependent strand annealing were also present in the strain RSPS-4 genome: PolA (RradSPS_0202), which participates in the initial DNA synthesis-step, RecA, that ensures the maturation of the linear intermediates into full-size circular chromosomes and RadA (RradSPS_1965), a protein involved in the stabilization or processing of branched DNA molecules [69] and in the priming step in DNA strand elongation [66].

Homologs of the Ku-like complex proteins and DNA ligase IV complex, the most relevant proteins involved in non-homologous end-joining pathway were not found in RSPS-4 strain, suggesting that this repair pathway may not be functional.

Mismatch repair (MMR) is probably functional in RSPS-4 with gene copies of *mut*L (RradSPS_0036 and _0159), and *mut*S (RradSPS_0158). The absence of the gene encoding the MutH endonuclease, which participates in the recognition of GATC methylated sequences discriminating the DNA strand to be repaired, should not compromise the efficiency of this pathway, as this protein is also absent from several bacterial genomes [70]. No *dcm* or *dam* site specific methylase genes were detected, suggesting that strain RSPS-4 uses different proteins for strand recognition and incision to complete MMR. Homologs of *xse*A and *xse*B, encoding the subunits of a MMR exonuclease, were not detected.

A particular feature of the RSPS-4 DNA repair pathways was the absence of the LexA autoprotease, the repressor that controls the expression of the SOS regulon in *E. coli*. The gene could not be identified in the RSPS-4 genome, based on a nucleotide sequence search using the two *lex*A genes described for the type strain DSM 5868^T^. Furthermore, the absence of this gene was validated by PCR amplification, using the genes of *R. radiotolerans* strain DSM 5868^T^ as template to design specific *lex*A primers. The absence of this gene has been observed in bacteria from many genera, such as *Mycoplasma*, *Chlamydia*, *Borrelia*, *Helicobacter*, *Coxiella*, and the *Cyanobacteria* [71-73], and suggests alternative mechanisms to repair DNA damage in bacteria could be employed. In *Lactococcus lactis,* a LexA–independent SOS response is led by the HdiR protein [74]. Moreover, other mechanisms may act alone or synergistically with the SOS response in DNA damage repair. In the radio-resistant *D. radiodurans,* the two LexA homologs undergo RecA-dependent cleavage after DNA damage, however, no regulon under the control of LexA1 or LexA2 proteins has been identified to date [66]. In this organism, *recA* induction following gamma irradiation is not controlled by LexA1 or LexA2 but depends on a *Deinococcus* specific regulatory protein IrrE, also designated PprI, a positive effector that enhances the expression of some DNA repair genes following exposure to radiation [66].

In deinococci, additional genes have been identified for DNA repair of irradiation damage, however these were absent from strain RSPS-4. No homologs of *irr*E or *ppr*A were found [75], suggesting that *Rubrobacter* uses other pathways for the same mechanism. The *ddr*A, *ddr*B, *ddr*C and *ddr*D genes [76] are also absent. Their absence from the *Kineococcus radiotolerans* genome [77] suggests their specificity to the deinococci.

### Reactive oxygen species detoxification

Reactive oxygen species (ROS) including hydrogen peroxide, superoxide and hydroxyl radicals are toxic to cells due to their ability to damage DNA and specially proteins containing iron-sulfur clusters or sculpture atoms. In order to prevent the damage induced by ROS, cells have several mechanisms of response to oxidative stress. Strain RSPS-4 has a single gene for a manganese containing catalase (RradSPS_2184), whilst *kat*A, *kat*E and *kat*G were not detected [15,78]. Superoxide dismutase was also encoded as a single gene identified as *sod*A (RradSPS_0327), and was also dependent on manganese [79]. Peroxiredoxins, which reduce H_2_O_2_ to water, were encoded by six copies of alkyl hydroperoxide reductase subunit C/ Thiol specific antioxidant (AhpC/TSA) (RradSPS_0148, _0515,_ 988, _1124, _2530, _2650) while peroxidases such as BsaA were not detected. A single gene for thioredoxin reductase *trx*R (RradSPS_0074) [78] was detected in the genome, as well as a gene for the *trx*A (RradSPS_0519) and two for *grx*A redoxins (RradSPS_1230 and _3087), involved in redox balance [80]. A single gene for thiosulfate transferase (RradSPS_0885) [78] was also found in the genome. Although regulatory genes of the *oxy*R family were not detected, *lys*R (RradSPS_1021, _1060, _1715, _1856 and _2024), which activates the transcription of genes involved in peroxide metabolism and protection in *D. radiodurans* (*kat*G, *ahp*C, *ahp*F, and *dps*) were detected [80].

Manganese has been proposed to be an important mechanism for oxidative stress response. This ion is suggested to protect cytosolic proteins from ROS by replacing Fe^2+^ and other divalent cations such as Mg^2+^ or Cu^2+^ as cofactors, and by forming ROS-scavenging complexes with various metabolites that preserve enzyme quaternary structures [23]. In strain RSPS-4, the catalase and the superoxide dismutase genes are predicted to encode manganese-containing enzymes and two ABC-type Mn^2+^/Zn^2+^ transport systems were present in the genome sequence (RradSPS_1136, _1137 and _1138; RradSPS_2222, _2223 and _2224) [80].

### Compatible solutes and stress protection

Two compatible solutes, namely trehalose and mannosylglycerate, accumulate to high levels in *R. xylanophilus* and *R. radiotolerans* RSPS-4 [81,82], but the accumulation of these osmolytes is not dependent on the salt concentration of the growth medium as it is in so many halotorerant and halophilic organisms. Instead, the accumulation of these compatible solutes is constitutive indicating that the accumulation of these compatible solutes is most likely in response to any of several stress conditions that may affect the survival of the cells [81]. The genome sequence of *R. radiotolerans* RSPS-4 and *R. radiotolerans* DSM 5868^T^ possess many of the key genes for the synthesis of trehalose and mannosylglycerate. Strain RSPS-4 and DSM 5868^T^ possess identical mannosylphospglycerate synthases that are homologs of *R. xylanophilus* (*mpg*S, EU847586.1) (RradSPS_0500; Rrad_0501). The synthesis of trehalose could proceed via four pathways namely through the TpS/TpP (RradSPS_0264 and _0265; Rrad_0265 and _0266), the TreS (RradSPS_0192; Rrad_0194), the TreT (RradSPS_0753; Rrad_0753) or the TreY/TreZ pathways (RradSPS_0196 and _0195; Rrad_0198 and _0197), homologs of which are found in both *R. radiotolerans* strains. The TpS/TpP and the TreT pathways were examined in *R. xylanophilus* and both were involved in the synthesis of trehalose [82]. Trehalose is considered to be a solute involved in the protection of many biological structures under different stress conditions and is considered to be especially important under extreme desiccation [83].

### Comparisons with other fully sequenced genomes

The genome sequence of *R. radiotolerans* RSPS-4 was compared to the genome of *Rubrobacter radiotolerans* DSM 5868^T^. The genome structure was similar in the two strains, a major chromosome and 3 plasmids. However, the genome of strain DSM 5868^T^, with 3,398,074 bp and 3266 genes, is larger by 130,841 bp than the genome of the RSPS-4 genome. Alignment of the two genome nucleotide sequences with the tool MISHIMA [84] revealed the two genome sequences practically shared the same nucleotide sequence, the same order, orientation and synteny. The main differences resided in chromosome 1 and plasmid 1. In chromosome 1, strain DSM 5868^T^ had an extra 87,086 bp segment of 70 genes (Table S2). This segment encodes a putative prophage, flanked by an integrase in the first position (Rrad_2453), and transposases at the other end (Rrad_2527 and _2528). Plasmid 1 of DSM 5868^T^ contained a segment of 55 genes that was only observed in this strain (Table S3). This 56,691 bp segment was flanked at one end by a resolvase (Rrad_2844) but no enzyme related to mobile elements was observed at the other end. Strain RSPS-4 also had a genome segment that was absent from the DSM 5868^T^ strain. This was a segment of 11 genes, corresponding to 11,639 bp, in plasmid 1 (Table S4). This segment harbors two transposases (RradSPS_2871 and _2872) and a recombinase (RradSPS_2873), although they are positioned in the middle of the segment and may be the remnant of the insertion of mobile elements.

Apart from the proteins related to mobile elements, the two additional segments present in the type strain have several transcriptional regulators (Table S2 and S3). *Lex*A was present in two copies, one in the chromosomal segment (Rrad_2461) and the other in plasmid 1 (Rrad_3065). The location of this gene within a mobile region may explain its absence from the RSPS-4 strain. Other transcriptional regulators were identified in these genome segments; the transcriptional regulator of the XRE family (Rrad_2455 and _3061), which acts as a repressor–like protein of several phages [85], ArsR (Rrad_2469), which participates in the stress response to heavy metals [86] and LuxR (Rrad_2505 and _2856) and TetR (Rrad_2866 and _2871), which are involved in diverse pathways such as those encoding virulence factors and antibiotics biosynthesis [87], the control of multidrug efflux pumps, and the response to osmotic stress or toxic chemicals [88]. The extra segments present in DSM 5868^T^ genome were populated by hypothetical or conserved hypothetical genes, 32 in the chromosome 1, and 29 in plasmid 1 (Table S2 and S3). The segments additionally encoded transporters involved in heavy metal homeostasis (such as heavy metal translocating P-type ATPase, (Rrad_2468)), proteins involved in lipid biosynthesis (such as 4'-phosphopantetheinyl transferase), proteins involved in the type II/III secretion system (Rrad_2515, _2516 and _2853) and several enzymes involved in the respiratory chain (Rrad_2470-2477) (Table S2). Most genes in the extra segments of type strain were actually also encoded in other regions of the genome, suggesting that the duplication of certain genes may provide the DSM 5868^T^ strain with increased ability to respond to stress conditions.

## Conclusion

The complete genome sequence and annotation of *Rubrobacter radiotolerans* strain RSPS-4 was hereby presented. The genome comprises 1 chromosome and 3 circular plasmids which together represent an organism of approximately 3.2 Mb. The genome sequence encodes for several key genes involved in the mechanisms of DNA repair, in response to oxidative stress and in the production of compatible solutes. However, some of the described pathways are not complete and await further studies to fully understand the mechanisms behind the RSPS-4 extreme resistance to radiation.
